# Supplementary material for: Laser-driven resonant soft-X-ray scattering for probing picosecond dynamics of nanometre-scale order
Source: Light Sci Appl. 2025 Dec 2;14:394. doi: 10.1038/s41377-025-02088-2 (PMC12669721; doi:10.1038/s41377-025-02088-2)
Supplement: Supplementary file 1 — Supplementary Information [file 41377_2025_2088_MOESM1_ESM.pdf]

Supplementary Information for

Laser-driven resonant soft-X-ray scattering for probing picosecond dynamics of nanometre-scale order

Leonid Lunin[1†], Martin Borchert[1†], Niklas Schneider[1], Konstanze Korell[1], Michael Schneider[1], Dieter Engel[1], Stefan Eisebitt[1,2], Bastian Pfau[1*], Daniel Schick[1*]

[1]Max-Born-Institut für Nichtlineare Optik und Kurzzeitspektroskopie, Max-Born-Straße 2A, 12489 Berlin, Germany.
[2]Technische Universität Berlin, Institut für Optik und Atomare Physik, Straße des 17. Juni 135, 10623 Berlin, Germany.

*Corresponding author(s). E-mail(s): pfau@mbi-berlin.de; schick@mbi-berlin.de;
†These authors contributed equally to this work

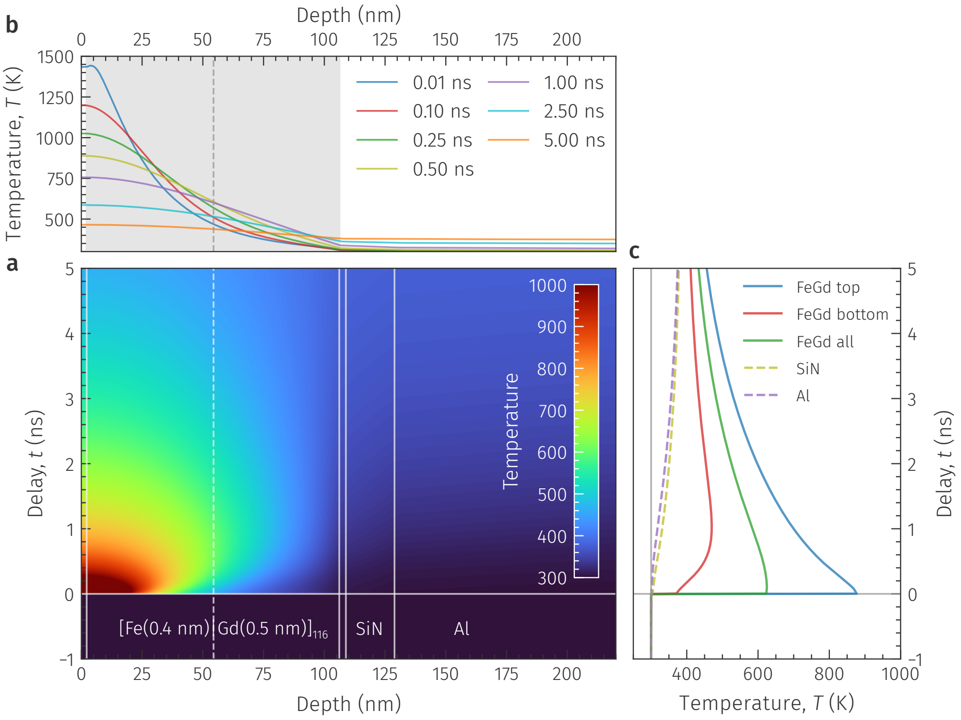

Fig. S1 One-dimensional heat diffusion simulation after laser excitation of the investigated FeGd heterostructure. Details of the simulations are described in the Method section. Values for the electronic and phononic thermal conductivities of FeGd are taken from Ref. [1]. **a**, Time-dependent temperature distribution within the entire sample (cap layer, magnetic multilayer, SiN substrate, Al heat sink). The vertical solid grey lines indicate the top and bottom of the actual magnetic multilayer, and the vertical dashed grey line its centre dividing the layer in a "top" and "bottom" part (see panel c). **b**, Line-outs of the spatial temperature distribution for different pump-probe delays t as indicated. **c**, Average temperature of different regions of the sample heterostructure as function of the pump-probe delay t.

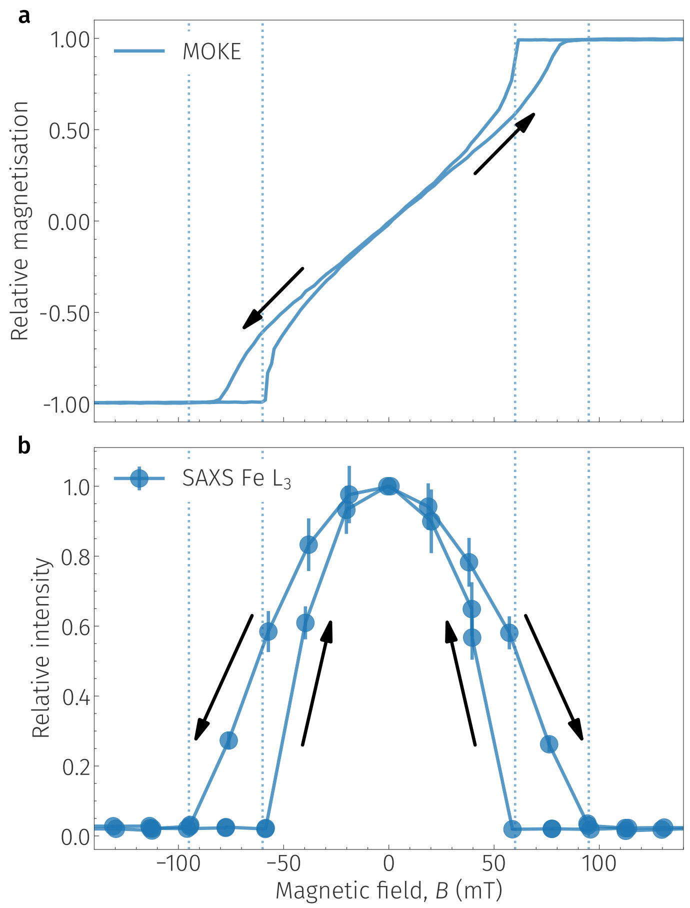

Fig. S2 Hysteresis scan of the investigated FeGd heterostructure. The black arrows indicate the scan direction of the applied magnetic field, B. **a**, Magneto-optical Kerr-effect (MOKE) measurement of the net out-of-plane magnetisation. In remanence, no net magnetisation is detectable as the contributions from oppositely magnetised domains cancel out. At larger fields ($B > 75\,\mathrm{mT}$) all domains align parallel into ferromagnetic saturation. **b**, The SAXS peak intensity at the Fe L_3 absorption edge exhibits maximum contrast approximately in remanence. The scattering intensity vanishes at high fields when the domain structure disappears in ferromagnetic saturation.

References

[1] Hopkins, P. E., Ding, M. D. & Poon, J. Contributions of electron and phonon transport to the thermal conductivity of GdFeCo and TbFeCo amorphous rare-earth transition-metal alloys. *Journal of Applied Physics* **111**, 103533 (2012). URL https://pubs.aip.org/jap/article/111/10/103533/369647/Contributions-of-electron-and-phonon-transport-to.
